# Dihydromyricetin attenuates fibrosis-associated features of hypertrophic scar with accompanying changes in PI3K/AKT/mTOR-related signaling

**DOI:** 10.3389/fphar.2026.1862651

**Published:** 2026-08-12

**Authors:** Peng Cao, Ao Shi, Yunwei Wang, Shuoyi Hui, Guozhong Lyu

**Affiliations:** 1 Burn & Trauma Treatment Center, Affiliated Hospital of Jiangnan University, Wuxi, China; 2 Wuxi School of Medicine, Jiangnan University, Wuxi, China; 3 Engineering Research Center of the Ministry of Education for Wound Repair Technology, Jiangnan University, Affiliated Hospital of Jiangnan University, Wuxi, China; 4 Department of Burn Plastic and Wound Repair Surgery, The Second Hospital of Lanzhou University, Lanzhou, China; 5 Department of Orthodontics, State Key Laboratory of Oral & Maxillofacial Reconstruction and Regeneration, National Clinical Research Center for Oral Diseases, Shaanxi Key Laboratory of Orthodontics, School of Stomatology, The Fourth Military Medical University, Xi’an, China; 6 National Emergency Medical Research Center, Emergency General Hospital, Beijing, China

**Keywords:** dihydromyricetin, fibrosis, hypertrophic scar, hypertrophic scar fibroblasts, network pharmacology, PI3K/AKT/mTOR signaling

## Abstract

This study investigated whether dihydromyricetin (DHM) attenuates fibrosis-associated features in hypertrophic scar (HS) and whether these effects are accompanied by changes in PI3K/AKT/mTOR-related signaling. Network pharmacology identified overlapping targets between DHM and HS, and subsequent protein–protein interaction and enrichment analyses highlighted AKT1 as a candidate hub target potentially associated with PI3K/AKT/mTOR-related signaling. Molecular docking and 50-ns molecular dynamics simulation further indicated a stable predicted interaction pattern between DHM and AKT1. For experimental validation, hypertrophic scar tissues, paired normal skin tissues, hypertrophic scar fibroblasts (HSFs), and paired normal skin fibroblasts (NSFs) were examined. Compared with paired normal controls, HS tissues and HSFs showed increased expression of collagen I, collagen III, and α-SMA. Untreated HSFs also exhibited higher basal p-AKT/AKT and p-mTOR/mTOR ratios than paired NSFs. In HSFs, DHM reduced the CCK-8 viability signal and the expression of fibrosis-related markers, and these changes were accompanied by decreased phosphorylation of AKT and mTOR. Live/dead staining did not show an obvious increase in PI-positive dead cells after 15 μM DHM treatment, suggesting that the decrease in CCK-8 signal may reflect reduced metabolic activity and growth inhibition rather than extensive acute cell death. Treatment with LY294002 alone and combined treatment with DHM and LY294002 further supported the involvement of PI3K/AKT/mTOR-related signaling in the regulation of fibrosis-associated features in HSFs. In a rabbit ear hypertrophic scar model, DHM improved gross scar appearance, reduced the scar elevation index, decreased the collagen-positive area fraction in Masson’s trichrome-stained sections, and alleviated histopathological alterations. Together, these findings indicate that DHM attenuated fibrosis-associated features of HS *in vitro* and *in vivo*. The observed effects were accompanied by changes in PI3K/AKT/mTOR-related signaling, supporting the involvement of the PI3K/AKT/mTOR signaling cascade. However, the current experimental design cannot confirm a definitive causal regulatory relationship. DHM may represent a potential anti-scar candidate and warrants further mechanistic and translational investigation.

## Highlights

- DHM attenuates fibrosis-associated features of hypertrophic scar *in vitro* and *in vivo*.
- AKT1 was prioritized as a hub target candidate potentially associated with DHM and HS.
- HSFs showed higher basal p-AKT/AKT and p-mTOR/mTOR ratios than paired NSFs.
- DHM reduced fibrosis-related markers with accompanying changes in PI3K/AKT/mTOR-related phosphorylation in HSFs.
- DHM reduced SEI and collagen-positive area fraction in rabbit ear hypertrophic scars.

## 1 Introduction

Hypertrophic scar (HS) is a common pathological outcome of cutaneous wound healing and is characterized by excessive fibroproliferation and extracellular matrix (ECM) deposition during tissue repair. Clinically, HS often presents as raised, erythematous, or hyperpigmented lesions accompanied by stiffness, pruritus, pain, and traction discomfort. In severe cases, it may cause contractures and functional limitations, thereby impairing quality of life and rehabilitation. HS commonly occurs after burns, trauma, and surgical injury and remains a major clinical challenge because of its high recurrence rate and the limited long-term efficacy of current treatments (Murakami and Shigeki, 2024; Mony et al., 2023).

Normal wound healing is a tightly regulated process involving inflammation, proliferation, and remodeling. When this process becomes dysregulated, persistent inflammation and sustained fibroblast activation can promote excessive collagen deposition, myofibroblast differentiation, and aberrant scar remodeling (Mony et al., 2023). Multiple signaling pathways, including transforming growth factor-β (TGF-β)/Smad, mitogen-activated protein kinase (MAPK), and phosphoinositide 3-kinase/protein kinase B/mammalian target of rapamycin (PI3K/AKT/mTOR), have been implicated in the pathogenesis of HS (Mony et al., 2023; Zhao et al., 2024; Dong et al., 2024). Among these pathways, TGF-β/Smad signaling is widely recognized as a central profibrotic pathway that promotes myofibroblast differentiation and ECM accumulation. Because fibrotic remodeling is not regulated by a single linear pathway, PI3K/AKT/mTOR signaling may interact with TGF-β/Smad-related responses and contribute to fibroblast survival, proliferation, collagen synthesis, and maintenance of the profibrotic phenotype (Zhao et al., 2024; Dong et al., 2024). Therefore, examining PI3K/AKT/mTOR-related changes may help clarify an important component of the signaling network associated with HS fibrosis. However, current clinical management still mainly relies on surgery, corticosteroid injection, laser therapy, and silicone-based treatment, all of which are limited by variable efficacy, recurrence, or adverse effects (Murakami and Shigeki, 2024). Accordingly, the identification of effective pharmacological interventions for HS remains of considerable interest.

Natural bioactive compounds have attracted increasing attention in the treatment of inflammation- and fibrosis-related disorders because of their broad biological activities and relatively favorable biocompatibility (Zhang et al., 2024). Dihydromyricetin (DHM), a natural flavonoid isolated from *Ampelopsis grossedentata* and other plants, has shown anti-inflammatory and anti-fibrotic activities in several preclinical settings (Wang et al., 2024; He et al., 2025). Previous studies suggest that DHM can modulate oxidative stress, inflammatory responses, and abnormal cellular activity, and may reduce fibroblast activation and collagen accumulation in fibrosis-related conditions (Wang et al., 2024; He et al., 2025). These findings raise the possibility that DHM may also exert anti-fibrotic effects in HS, although its role in hypertrophic scar formation and remodeling has not been systematically investigated.

Among the signaling pathways involved in fibrosis, PI3K/AKT/mTOR signaling has been widely linked to fibroblast activation, collagen synthesis, cell survival, and metabolic regulation (Zhao et al., 2024; Dong et al., 2024). Abnormal activation of this pathway has been reported in pathological tissue repair, suggesting that it may also contribute to HS development (Zhao et al., 2024; Dong et al., 2024). Given its potential interaction with other profibrotic pathways, including TGF-β/Smad signaling, PI3K/AKT/mTOR may represent a biologically relevant signaling axis for investigating the cellular responses of hypertrophic scar fibroblasts (HSFs). Whether DHM affects HS-associated fibrotic activity in relation to this pathway, however, remains unclear.

In recent years, network pharmacology, molecular docking, and molecular dynamics simulation have become useful tools for exploring potential targets and pathway associations of natural compounds. Rather than serving as definitive mechanistic proof, these approaches are particularly valuable for hypothesis generation and for guiding subsequent experimental validation (Lee et al., 2025; Panossian, 2025). When combined with cellular and animal experiments, they can provide an integrated view of the possible pharmacological actions of candidate compounds. Nevertheless, computational predictions require experimental validation and should be interpreted as supportive evidence rather than direct proof of target engagement or pathway causality.

**SCHEME 1:**
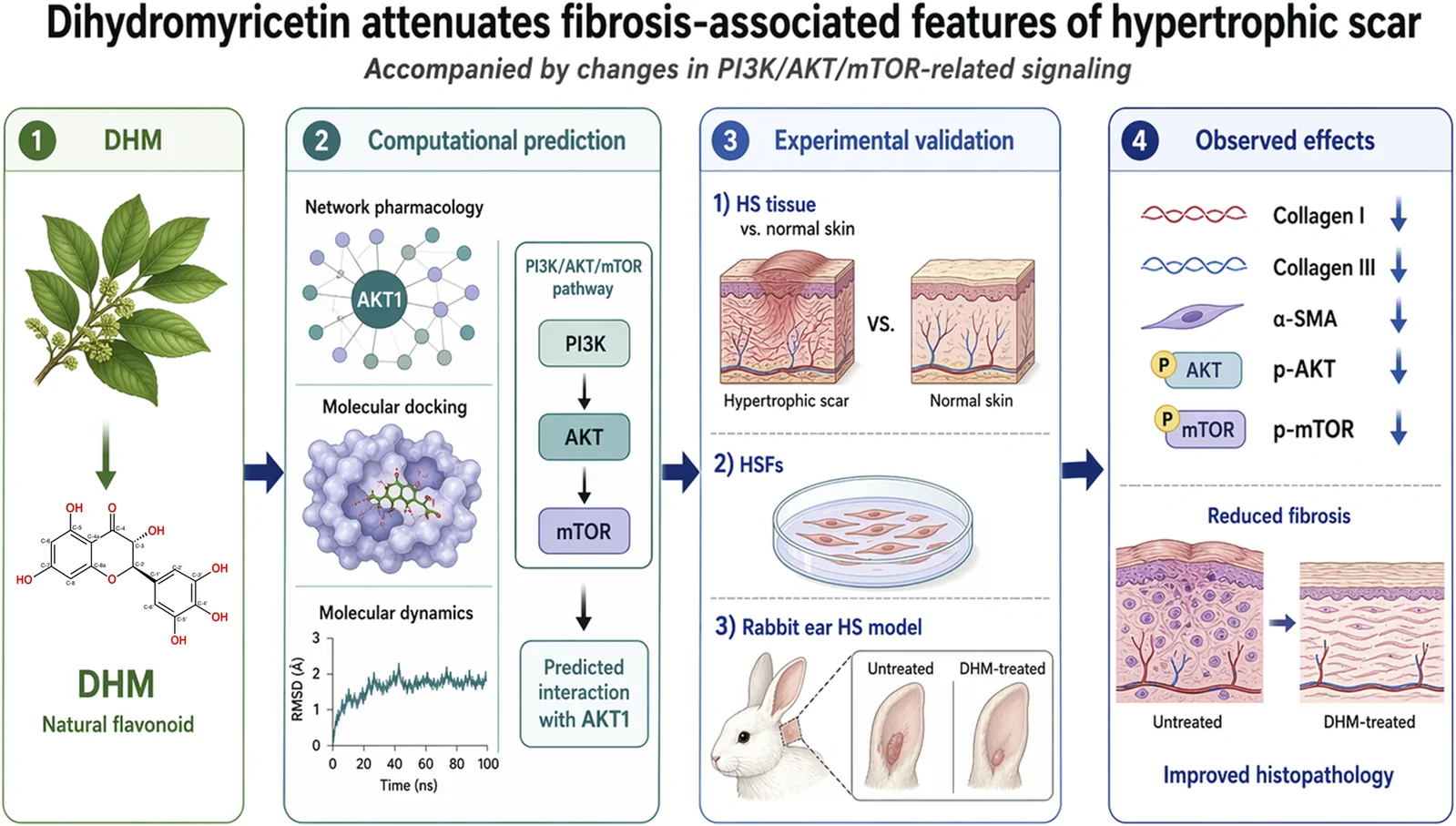
Schematic overview of DHM-associated attenuation of hypertrophic scar-related fibrotic features accompanied by changes in PI3K/AKT/mTOR-related signaling. Conceptual illustrations in Scheme 1, except for the DHM chemical structure, were generated using Kimi AI (Moonshot AI) and subsequently reviewed and edited by the authors.

Based on these considerations, the present study employed an integrated computational and experimental strategy to investigate whether DHM attenuates fibrosis-associated features of HS and whether these effects are accompanied by changes in PI3K/AKT/mTOR-related signaling. Network pharmacology, molecular docking, and molecular dynamics simulation were used to generate hypotheses regarding candidate targets and pathway associations, which were further examined in HS tissues, hypertrophic scar-derived fibroblasts, paired normal skin fibroblasts, and a rabbit ear hypertrophic scar model. Through this integrated approach, this study aimed to evaluate the anti-scar potential of DHM while providing preliminary evidence supporting the involvement of PI3K/AKT/mTOR-related signaling. However, this study was not designed to establish a definitive causal regulatory relationship between DHM and the PI3K/AKT/mTOR signaling cascade (Scheme 1).

## 2 Materials and methods

### 2.1 Materials and reagents

Dihydromyricetin (DHM, purity ≥98%, CAS: 27200-12-0) was purchased from Macklin Biochemical Co., Ltd. (Shanghai, China). The PI3K inhibitor LY294002 (CAS: 154447-36-6) was obtained from MedChemExpress (Monmouth Junction, NJ, United States). DHM and LY294002 were dissolved in dimethyl sulfoxide (DMSO) to prepare 50 mM stock solutions and stored at −20 °C protected from light. Working solutions were freshly diluted in culture medium before each experiment, and the final concentration of DMSO was maintained at ≤0.1% (v/v) in all cell treatment groups. DMEM, trypsin, fetal bovine serum (FBS), and penicillin-streptomycin were purchased from Gibco (United States). Cell Counting Kit-8 (CCK-8) was obtained from APExBIO (United States). The Calcein-AM/PI Double Staining Kit used for live/dead staining was purchased from Beyotime Biotechnology (Shanghai, China). TRIzol reagent was purchased from Invitrogen (United States), while the PrimeScript RT reagent kit and SYBR Premix Ex Taq II kit were purchased from TaKaRa (Japan). RIPA lysis buffer was obtained from Solarbio (China), and the BCA protein assay kit was purchased from Beyotime Biotechnology (Shanghai, China).

The primary antibodies used in this study included anti-Collagen I (Abcam, United Kingdom), anti-Collagen III (Abcam, United Kingdom), anti-AKT1 and anti-phospho-AKT (Ser473) (Cell Signaling Technology, Danvers, MA, United States), anti-α-SMA (Cell Signaling Technology, United States), anti-mTOR (Cell Signaling Technology, United States), anti-phospho-mTOR (Cell Signaling Technology, United States), and anti-β-actin (Cell Signaling Technology, United States). Cy3- or Alexa Fluor 488-conjugated secondary antibodies for immunofluorescence were purchased from Cwbio (China), and DAPI was obtained from Sigma. Hematoxylin and eosin (H&E) staining kit and Masson’s trichrome staining kit were purchased from Solarbio (China). Unless otherwise specified, all other reagents were of analytical grade.

### 2.2 Network pharmacology analysis

#### 2.2.1 Acquisition of the chemical structure of DHM

The standardized chemical structure and SMILES information of DHM were obtained from the PubChem database by searching the term “dihydromyricetin” (National Center for Biotechnology Information, 2025). The two-dimensional and three-dimensional structure files were downloaded for subsequent network pharmacology analysis, molecular docking, and molecular dynamics simulation.

#### 2.2.2 Prediction of potential targets of DHM

Potential targets of DHM were retrieved using the keyword “dihydromyricetin” from the Traditional Chinese Medicine Systems Pharmacology Database and Analysis Platform (TCMSP), SwissTargetPrediction, and the Comparative Toxicogenomics Database (CTD). In SwissTargetPrediction, the species was restricted to *Homo sapiens*, and only targets with a predicted probability >0 were retained (Ru et al., 2014; Davis et al., 2023; Daina et al., 2019). Because different target-prediction databases use different algorithms and scoring systems, targets from multiple databases were integrated to improve target coverage and reduce database-specific bias. Targets from different databases were merged, deduplicated, and standardized using the UniProt database to obtain the final set of putative DHM-related targets (UniProt, 2023). Only targets that could be successfully mapped to official human gene symbols in UniProt were retained for subsequent analysis.

#### 2.2.3 Identification of hypertrophic scar-related targets

The keyword “hypertrophic scar” was used to retrieve disease-related targets from the GeneCards, DisGeNET, and OMIM databases (Amberger et al., 2019; Piñero et al., 2020; Stelzer et al., 2016). The species was restricted to *H. sapiens* where applicable. Because these disease databases differ in annotation sources and scoring methods, disease-related targets were integrated across databases to improve coverage of HS-associated genes. The retrieved targets were integrated and deduplicated to generate the HS-related target set. Targets that could not be standardized to official human gene symbols were excluded.

#### 2.2.4 Identification of intersecting targets and network construction

The intersecting targets between DHM-related targets and HS-related targets were identified using an online Venn analysis tool (Ol and iveros, 2015). DHM and the intersecting targets were then imported into Cytoscape software to construct a drug-target network (Otasek et al., 2019; Shannon et al., 2003). The intersecting targets were further submitted to the STRING database to construct a protein-protein interaction (PPI) network, with the species limited to *H. sapiens* and the minimum interaction confidence set to 0.40, corresponding to the medium-confidence interaction threshold recommended by STRING (Szklarczyk et al., 2023). The resulting network was exported and visualized in Cytoscape.

#### 2.2.5 Hub target analysis and enrichment analysis

The CytoHubba plugin in Cytoscape was used to rank nodes in the PPI network using four topological algorithms: Degree, Betweenness, Maximum Clique Centrality (MCC), and Closeness (Shannon et al., 2003; Assenov et al., 2008; Chin et al., 2014). For each algorithm, the top 10 ranked genes were extracted. Genes that appeared simultaneously in the top 10 lists generated by all four algorithms were regarded as hub target candidates. Gene Ontology (GO) functional enrichment analysis and Kyoto Encyclopedia of Genes and Genomes (KEGG) pathway enrichment analysis were then performed for the intersecting targets using Metascape (Zhou et al., 2019). Enrichment terms with a P value < 0.05 were considered statistically significant and were used for pathway interpretation. Based on the enrichment results, PI3K/AKT/mTOR-related signaling was selected for subsequent experimental examination. AKT1 was prioritized as a candidate target because it was consistently identified as a hub gene across multiple CytoHubba algorithms, was directly involved in PI3K/AKT-related enrichment results, and showed biological relevance to fibroblast activation and fibrosis-associated signaling. Therefore, AKT1 was chosen as a candidate target for molecular docking and molecular dynamics simulation.

### 2.3 Molecular docking and molecular dynamics simulation

#### 2.3.1 Molecular docking

To assess the potential interaction between DHM and candidate target proteins, molecular docking was performed using the CB-Dock2 online platform (accessed 1 April 2026). In general, a lower docking score indicates a more favorable predicted interaction between the ligand and receptor. CB-Dock2 is an AutoDock Vina-based docking tool that can automatically identify potential binding cavities between ligands and receptors (Liu et al., 2021).

The three-dimensional structure of DHM was downloaded from PubChem in SDF format and uploaded to CB-Dock2 for hydrogenation and energy minimization. Based on the network pharmacology results, AKT1 was selected as a candidate receptor protein, and its three-dimensional crystal structure was downloaded from the RCSB Protein Data Bank using PDB ID: 8UW9 in PDB format (Berman et al., 2000). After receptor preprocessing, including removal of water molecules and addition of hydrogen atoms, molecular docking was conducted on the CB-Dock2 platform to evaluate the potential binding pattern between DHM and AKT1 and to identify the docking conformation with the lowest predicted binding energy. The ligand-receptor interaction pattern was visualized using PyMOL (version 3.0.3) and BIOVIA Discovery Studio Visualizer (version 4.5) (Schrödinger, 2025; Dassault, 2025). For exploratory comparison, molecular docking was also performed for the other hub target candidates, including EGF, EGFR, ESR1, IL6, MMP2, MMP9, and SRC, and the representative docking visualizations are provided in Supplementary Figure S1. These additional docking results were used as supplementary computational evidence, whereas AKT1 was prioritized for molecular dynamics simulation and subsequent experimental examination.

#### 2.3.2 Molecular dynamics simulation

To further evaluate the conformational behavior and predicted binding stability of the DHM-AKT1 complex, molecular dynamics simulation was performed for the optimal ligand-receptor complex. Simulations were carried out using YASARA software (version 10.3.16) under the AMBER14 force field (Biosciences, 2025). The simulation was conducted under the NPT ensemble, with temperature controlled using the Berendsen thermostat. Long-range electrostatic interactions were calculated using the particle-mesh Ewald (PME) method, and the cutoff distance for short-range van der Waals and Coulomb interactions was set to 8 Å.

A cubic solvent box was constructed, with each boundary extending at least 20 Å beyond the ligand-protein complex, and periodic boundary conditions were applied. The system was neutralized with 0.9% NaCl solution and equilibrated at 298 K and 1 bar. Initial energy minimization was performed using simulated annealing combined with the steepest descent method. The simulation time step was set to 1.25 fs, trajectory data were saved every 100 ps, and the total simulation time was 50 ns. After the simulation, the trajectory files were analyzed for root mean square deviation (RMSD), root mean square fluctuation (RMSF), and system energy changes to assess the dynamic stability of the complex. In addition, binding free energy was estimated from the simulation trajectory using the MM/PBSA-like method implemented in YASARA.

### 2.4 Collection of clinical tissue samples, fibroblast isolation and culture, and identification

#### 2.4.1 Source of clinical tissue samples

From January 2024 to December 2024, hypertrophic scar tissues (HS tissues) and paired normal full-thickness skin tissues (NS tissues) were collected from patients undergoing surgery at the Department of Burn and Skin Surgery, the First Affiliated Hospital of Air Force Medical University. A total of six patients were included, comprising three males and three females, aged 12–43 years, with scar duration ranging from 6 to 10 months. Patients were included if they had clinically diagnosed hypertrophic scars and required surgical scar excision. Patients with keloids, active local infection, systemic inflammatory or autoimmune diseases, malignant tumors, or prior local anti-scar treatment, including corticosteroid injection, radiotherapy, laser therapy, or other anti-scar medication, were excluded. The HS sites included the shoulder, chest, neck, back, and buttock, while paired normal skin samples were obtained from the upper arm, abdomen, and back. The clinical characteristics of the patients are shown in Supplementary Table S1. Written informed consent was obtained from all patients prior to surgery for the use of resected tissues in this study. For the participant younger than 18 years of age, written informed consent was obtained from the legal guardian in accordance with institutional ethical requirements. This study was approved by the Ethics Committee of the First Affiliated Hospital of Air Force Medical University (Approval No. 20213138). The collected tissues were used for tissue-level validation and fibroblast isolation.

#### 2.4.2 Fibroblast isolation and culture

The dermal layers of HS tissues and paired NS tissues were collected, and fibroblasts were isolated using the explant culture method. Tissue samples were minced into small pieces and seeded into culture flasks containing DMEM complete medium supplemented with 10% FBS, 100 U/mL penicillin, and 100 μg/mL streptomycin. Cells were cultured in a humidified incubator at 37 °C with 5% CO_2_. After fibroblasts migrated out of the tissue explants and were stably passaged, hypertrophic scar fibroblasts (HSFs) and paired normal skin fibroblasts (NSFs) were obtained for subsequent experiments. Cells from passages 3–6 were used in the experiments.

#### 2.4.3 Fibroblast identification

Immunofluorescence staining for vimentin was performed to confirm the fibroblast phenotype. After fixation, cells were incubated with an anti-vimentin primary antibody, followed by a fluorescent secondary antibody. Nuclei were counterstained with DAPI, and cells were observed under a fluorescence microscope.

#### 2.4.4 Validation of the basic phenotypes of tissues and cells

RT-qPCR was used to detect the expression levels of Collagen I, Collagen III, and α-SMA in HS tissues and paired NS tissues, as well as in HSFs and paired NSFs, in order to confirm the fibrotic characteristics of the collected tissues and isolated cells. In addition, Western blotting was performed to compare the basal activation status of PI3K/AKT/mTOR-related signaling between untreated HSFs and paired NSFs by detecting p-AKT, AKT, p-mTOR, and mTOR protein levels. The ratios of p-AKT/AKT and p-mTOR/mTOR were calculated to evaluate basal pathway activation.

### 2.5 Cell experiments

#### 2.5.1 CCK-8 assay for the effects of DHM on HSF viability

HSFs were seeded into 96-well plates at a density of 5 × 10^3^ cells/well and allowed to adhere overnight. The cells were then treated with different concentrations of DHM for 24 h. DHM was dissolved in DMSO, and an equal volume of DMSO vehicle was added to the control wells to ensure the same final DMSO concentration (≤0.1%, v/v) in all groups. After treatment, CCK-8 working solution was added to each well, followed by incubation at 37 °C for 1 h. Absorbance was measured at 450 nm using a microplate reader. The CCK-8 assay was used to assess metabolic cell viability. The concentrations used in subsequent experiments were selected according to the cell viability results.

#### 2.5.2 Live/dead staining

To further evaluate whether the selected working concentration of DHM induced overt cell death, HSFs were treated with vehicle or 15 μM DHM for 24 h. Live/dead staining was performed using a Calcein-AM/PI Double Staining Kit according to the manufacturer’s instructions. Briefly, after treatment, cells were washed with PBS and incubated with Calcein-AM and propidium iodide (PI) staining solution at 37 °C in the dark. Live cells were visualized by green Calcein-AM fluorescence, whereas dead cells were identified by red PI fluorescence. Images were captured using a fluorescence microscope.

#### 2.5.3 Cell grouping and treatment

According to the results of the cell viability assay, HSFs were treated with DHM at 0, 5, 10, and 20 μM for 24 h in the dose-dependent experiments. Based on the cell viability results and preliminary observations of fibrosis-related marker changes, 15 μM DHM was selected as the working concentration for subsequent pathway-related experiments. For pathway-related experiments, cells were divided into four groups: vehicle control, DHM, LY294002, and DHM + LY294002. Cells in the DHM group were treated with 15 μM DHM for 24 h. Cells in the LY294002 group were treated with 10 μM LY294002 for 24 h. In the DHM + LY294002 group, cells were pretreated with LY294002 at a final concentration of 10 μM for 1 h before DHM treatment. An equal volume of DMSO was added to all groups as a vehicle control to ensure the same final DMSO concentration (≤0.1%, v/v). After 24 h of treatment, cells were collected for RT-qPCR, Western blotting, and immunofluorescence analyses.

#### 2.5.4 RT-qPCR

Total RNA was extracted from each group using TRIzol reagent. After RNA concentration and purity were determined, reverse transcription was performed according to the manufacturer’s instructions to synthesize cDNA. Real-time quantitative PCR was then conducted using GAPDH as the internal reference gene, and relative gene expression was calculated by the 2^-ΔΔCt^ method. Target genes included Collagen I, Collagen III, α-SMA, AKT1, and mTOR. Primer sequences are listed in Supplementary Table S2.

#### 2.5.5 Western blotting

Total protein was extracted from each group using RIPA lysis buffer, and protein concentrations were measured using the BCA protein assay kit. Equal amounts of protein were separated by SDS-PAGE and transferred onto PVDF membranes. After blocking, membranes were incubated overnight at 4 °C with primary antibodies against p-AKT, AKT, p-mTOR, mTOR, Collagen I, Collagen III, α-SMA, and β-actin. On the following day, membranes were incubated with HRP-conjugated secondary antibodies and visualized using enhanced chemiluminescence (ECL). Band intensities were analyzed using ImageJ software. Phosphorylated proteins were normalized to their corresponding total proteins, and fibrosis-related proteins were normalized to β-actin. β-actin was used as the loading control for semiquantitative analysis.

#### 2.5.6 Immunofluorescence staining

After treatment, HSFs were seeded onto coverslips. When the cells reached an appropriate density, they were fixed with 4% paraformaldehyde for 30 min. After washing with PBS, cells were permeabilized with 0.1% Triton X-100 for 10 min and blocked with 1% BSA. The cells were then incubated overnight at 4 °C with an anti-Collagen I primary antibody. On the following day, after washing, the cells were incubated with the corresponding fluorescent secondary antibody at 37 °C for 1 h in the dark. Nuclei were counterstained with DAPI, and images were captured under a fluorescence microscope. Fluorescence intensity was quantified using ImageJ software.

### 2.6 Animal experiments

#### 2.6.1 Experimental animals, establishment of the rabbit ear hypertrophic scar model, and DHM intervention

Six healthy New Zealand white rabbits of either sex, aged 4–6 months and weighing 2.0–2.5 kg, were used in this study. All rabbits had well-developed bilateral ears without obvious abnormalities. The animals were provided by the Experimental Animal Center of Air Force Medical University (Production License No. SCXK [Shaanxi] 2019-001). All animal procedures complied with institutional and national regulations for laboratory animal management and were approved by the Institutional Animal Care and Use Committee of Air Force Medical University (Approval No. IACUC-20220920).

The rabbits were housed individually under standard laboratory conditions for 1 week to acclimatize, fed a mixed artificial diet, and maintained at 17 °C–25 °C and 50%–75% relative humidity. Water was withheld for 8 h before surgery, and body weight was recorded. Anesthesia was induced by intravenous injection of 3% pentobarbital sodium (30 mg/kg) via the marginal ear vein. After anesthesia, the short hair on the ventral side of the rabbit ears was shaved using an electric clipper. The surgical area was disinfected three times with iodophor, covered with a sterile drape, and cleaned with gauze.

Four circular full-thickness skin wounds with a diameter of 1 cm were created on the ventral side of each ear while avoiding visible blood vessels. Thus, each rabbit had a total of eight wounds on both ears, and 48 wounds were created in six rabbits. The distance between adjacent wounds was at least 1.5 cm. A 1-cm biopsy punch was used to mark the wound positions. Full-thickness skin was excised along the marked line down to the perichondrium, and the skin was separated from the perichondrium to remove residual tissue from the wound bed. Hemostasis was achieved with sterile saline gauze compression, and erythromycin ointment was applied to the wound surface to prevent infection.

After recovery from anesthesia, the rabbits were returned to their cages and maintained under the original housing conditions. The wounds were left exposed until the hypertrophic scar model matured, and scar formation was evaluated on postoperative day 28 (Kloeters et al., 2007). Only wounds that developed mature raised scars by postoperative day 28 were included in the subsequent intervention and histological analyses. After hypertrophic scars had formed, intervention was performed using a paired design, with one ear of each rabbit assigned to the DHM group and the contralateral ear assigned to the PBS group. In the DHM group, DHM (50 μM) was injected intradermally into the scars, whereas the PBS group received an equal volume of PBS by the same route. The intervention was administered continuously for 4 weeks.

#### 2.6.2 Gross observation and model evaluation

The wound healing and scar formation process was observed on postoperative days 0, 7, 14, 21, and 28, and model establishment was assessed on day 28. The total number of wounds and the number of wounds showing raised scar formation were recorded, and the scar formation rate was calculated. After the intervention period, representative gross scar morphology was photographed and compared between groups in terms of scar elevation, thickness, color, and surface appearance.

#### 2.6.3 Histological analysis

At the end of the intervention, scar tissues from the paired PBS- and DHM-treated groups were harvested for histological examination. Paraffin-embedded samples were sectioned into 5-μm-thick serial slices, and a total of 15 sections per group were used for descriptive histological observation. Hematoxylin and eosin (H&E) staining was performed to evaluate epidermal thickening, inflammatory cell infiltration, fibroblast proliferation, and neovascularization. Masson’s trichrome staining was performed to assess collagen deposition and collagen fiber arrangement. For quantitative scar evaluation, the scar elevation index (SEI) was calculated from H&E-stained sections as the ratio of scar height to the height of adjacent normal skin. Collagen deposition was quantified on Masson’s trichrome-stained sections using ImageJ software, and the collagen-positive area fraction was calculated as the collagen-positive stained area divided by the total tissue area within the selected field. For SEI and collagen-positive area fraction analyses, three histological sections were analyzed for each treatment condition (n = 3 sections), and each section was used as one statistical unit.

### 2.7 Statistical analysis

Statistical analyses were performed using GraphPad Prism 11.0.0 software. Data are presented as mean ± SD. Unless otherwise stated, cell experiments were performed using three independent biological replicates. The exact n values and statistical methods used for each experiment are indicated in the corresponding figure legends.

For comparisons between hypertrophic scar tissues and paired normal skin tissues, as well as between hypertrophic scar fibroblasts (HSFs) and paired normal skin fibroblasts (NSFs) derived from the same donor, paired-samples t tests were used.

For SEI and collagen-positive area fraction analyses in the animal experiment, n refers to the number of analyzed histological sections rather than the number of rabbits or wounds. Each section was used as one statistical unit. Because PBS- and DHM-treated samples were obtained under a paired experimental design, paired-samples t tests were used for comparisons between the two treatment conditions.

For comparisons among three or more treatment groups in cell experiments, one-way ANOVA followed by Tukey’s multiple-comparisons test was used. Repeated-measures one-way ANOVA was applied when all treatment groups were measured within the same independent experiment. A two-sided P value < 0.05 was considered statistically significant. Statistical annotations were defined as follows: ns, not significant; *P < 0.05; **P < 0.01; ***P < 0.001; and ****P < 0.0001.

## 3 Results

### 3.1 Network pharmacology analysis suggested candidate targets and pathway associations involved in the effects of DHM on hypertrophic scar

**FIGURE 1:**
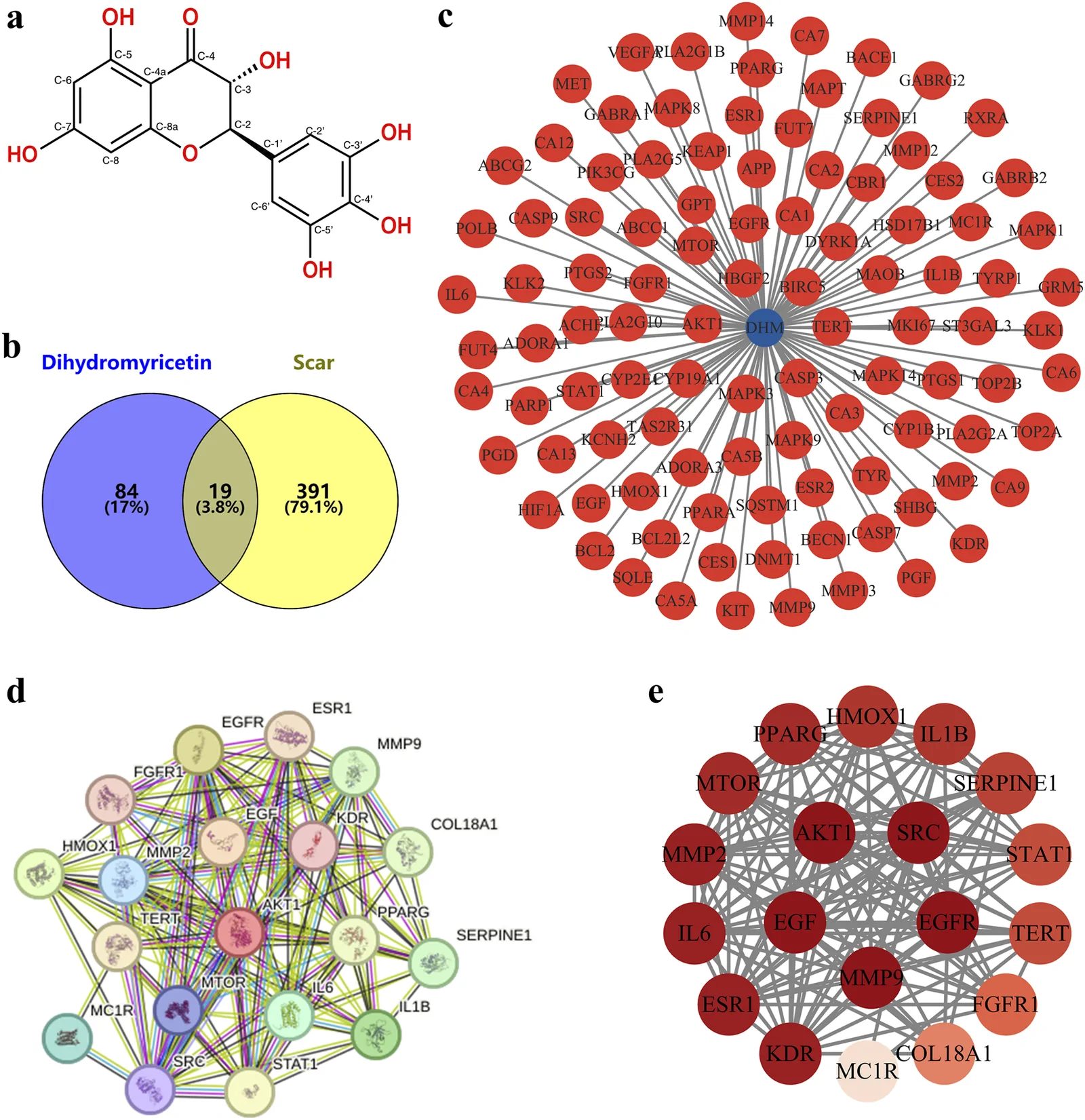
Network pharmacology-based prediction of potential targets of DHM in hypertrophic scar. **(a)** Chemical structure of DHM (C_15_H_12_O_8_). **(b)** Venn diagram showing overlapping targets between DHM and scar. **(c)** Drug-target network of DHM constructed in Cytoscape. **(d)** PPI network of common targets from STRING database. **(e)** PPI network diagram of 19 potential targets.

To explore the potential molecular basis by which DHM may affect hypertrophic scar formation, DHM-related targets and HS-related disease targets were first integrated, and their overlapping targets were identified by Venn diagram analysis (Figures 1a,b). A drug-target network was then constructed using Cytoscape, which showed that DHM was connected with multiple candidate targets, suggesting that the potential effects of DHM on hypertrophic scar may be related to multiple target candidates rather than a single isolated molecule (Figure 1c).

The overlapping targets were further imported into the STRING database to construct a protein-protein interaction (PPI) network. According to the network topology statistics, the PPI network contained 19 nodes and 132 edges, with an average node degree of 13.9, an average local clustering coefficient of 0.898, an expected number of 59 edges, and a PPI enrichment *P* value of 2.22 × 10^−16^ (Figures 1d,e). These findings suggest that the overlapping targets were functionally interconnected rather than randomly distributed.

**FIGURE 2:**
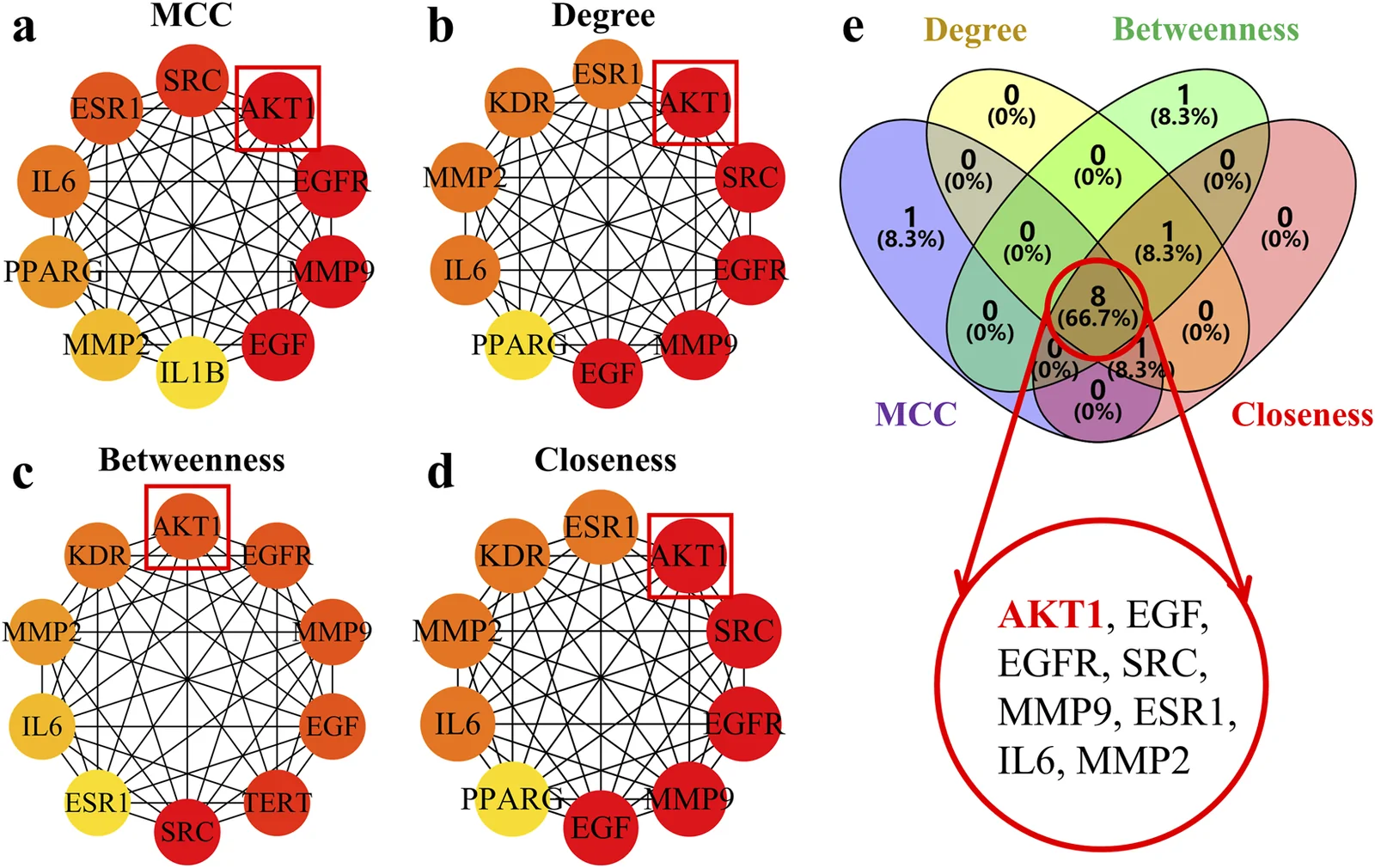
Identification of hub target candidates in the PPI network using CytoHubba. **(a)** Top 10 genes ranked by MCC. **(b)** Top 10 genes ranked by Degree. **(c)** Top 10 genes ranked by Betweenness. **(d)** Top 10 genes ranked by Closeness. **(e)** Venn diagram of common genes identified by four CytoHubba algorithms.

Subsequently, topological analysis of the PPI network was performed using the CytoHubba plugin based on four algorithms, including Degree, Betweenness, Maximum Clique Centrality (MCC), and Closeness. The results showed a high degree of overlap among the top-ranked genes identified by different algorithms. The common hub target candidates identified by the four algorithms included AKT1, EGF, EGFR, SRC, MMP9, MMP2, IL6, and ESR1 (Figures 2a–e). Among these candidates, AKT1 was prioritized for subsequent molecular docking, molecular dynamics simulation, and experimental examination because it was consistently ranked as a hub gene, was directly linked to the PI3K/AKT-related enrichment results, and was biologically relevant to fibroblast activation and fibrosis-associated signaling.

**FIGURE 3:**
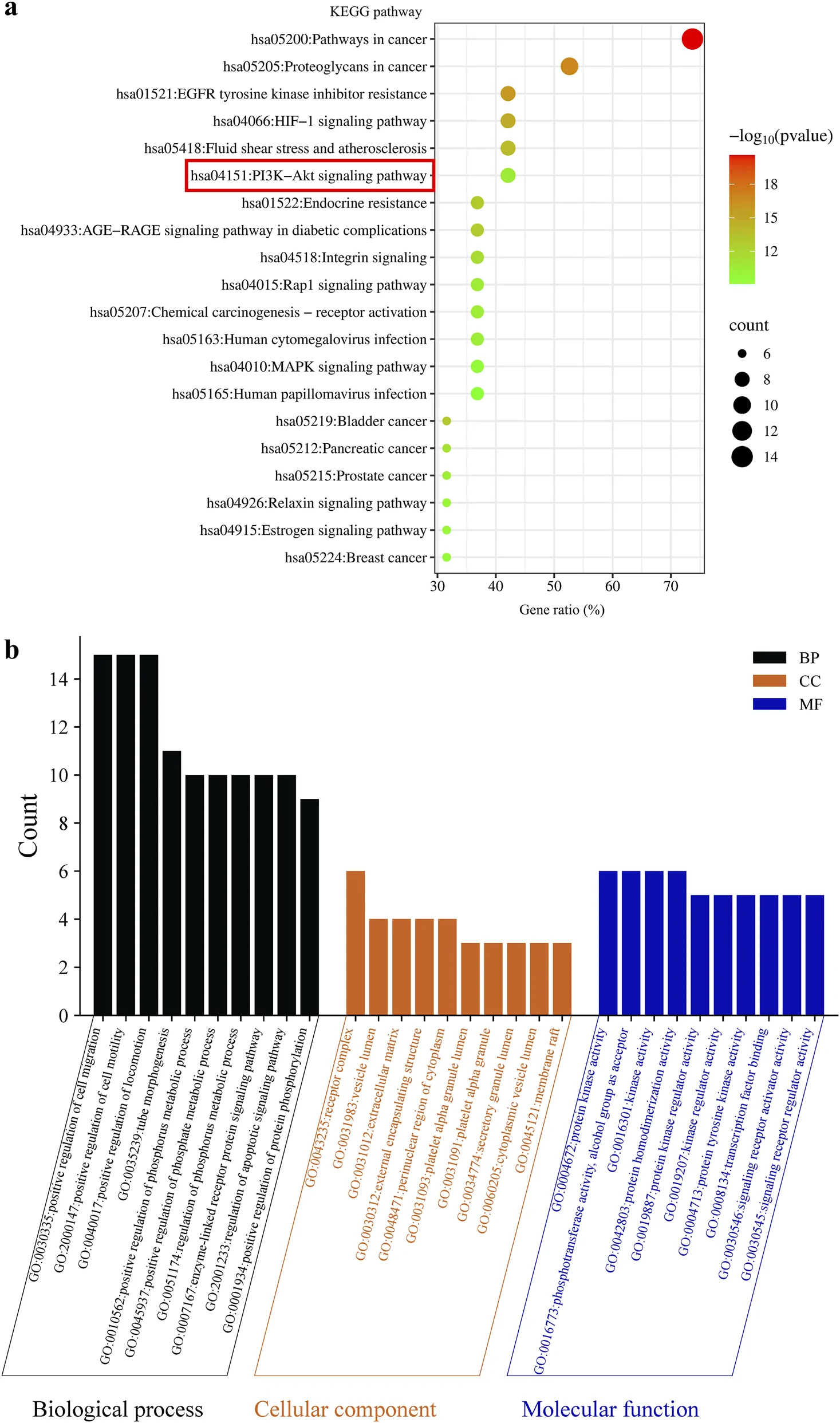
GO and KEGG enrichment analyses of the overlapping targets between DHM and hypertrophic scar. **(a)** KEGG pathway enrichment analysis showing enriched signaling pathways associated with the overlapping targets. The color gradient represents −log10(P value), and the bubble size represents the enriched gene count. **(b)** GO enrichment analysis showing enriched biological process (BP), cellular component (CC), and molecular function (MF) terms. Different colors indicate BP, CC, and MF categories, and the y-axis represents the enriched gene count.

GO enrichment analysis showed that the overlapping targets were mainly involved in biological processes related to cell proliferation, cell migration, extracellular matrix remodeling, and fibrosis regulation. KEGG enrichment analysis further indicated enrichment of PI3K/AKT-related signaling pathways (Figures 3a,b). Taken together, these results suggested that DHM may be associated with anti-hypertrophic scar effects and pointed to possible involvement of PI3K/AKT/mTOR-related signaling (Supplementary Figure S2).

### 3.2 Molecular docking and molecular dynamics simulation supported a predicted stable interaction pattern between DHM and AKT1

**FIGURE 4:**
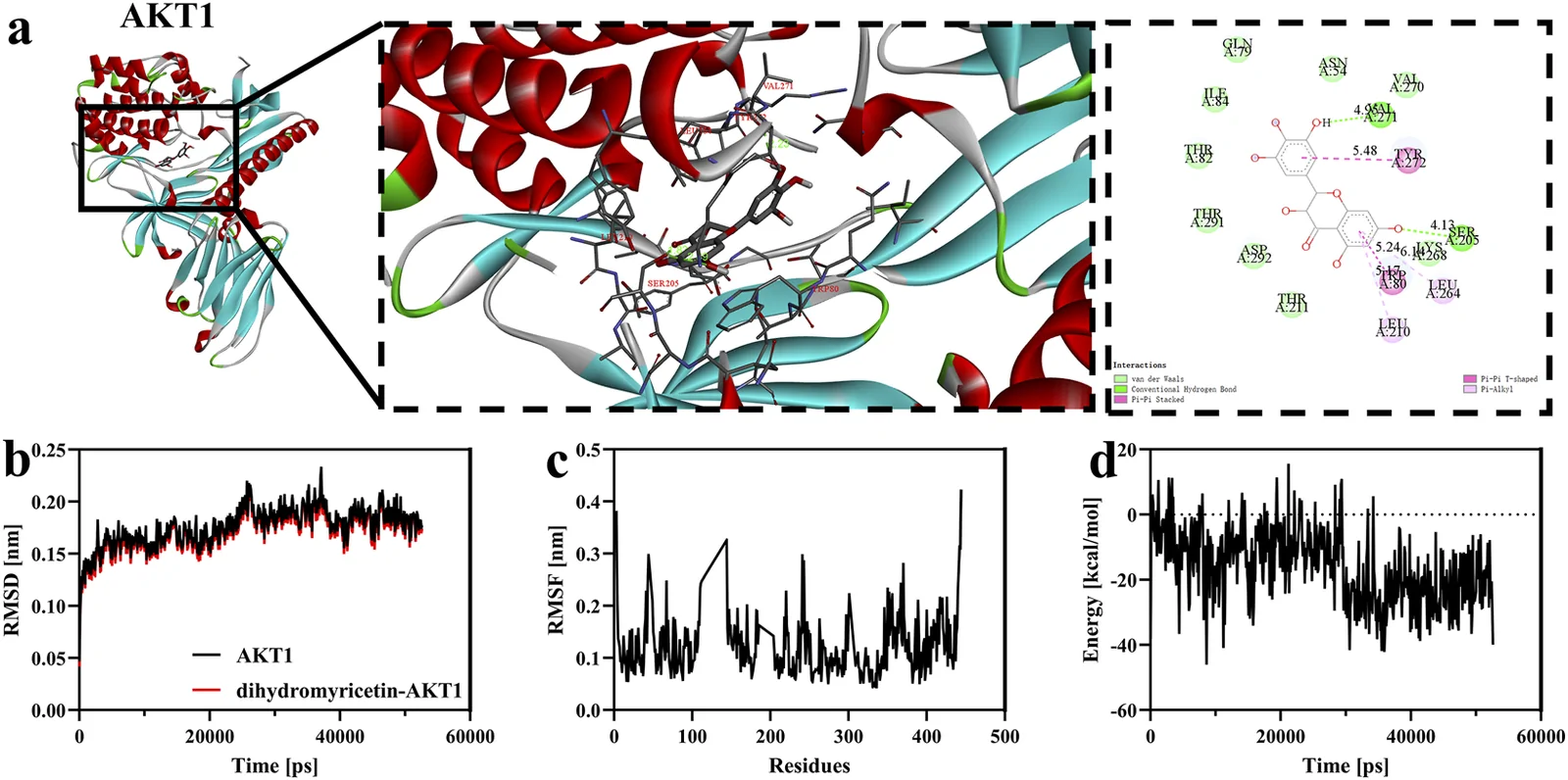
Molecular docking and molecular dynamics simulations of the DHM-AKT1 complex. **(a)** Molecular docking of DHM with AKT1 and visualization results. **(b)** Root mean square deviation (RMSD) curves showing overall structural stability during 50 ns simulations. **(c)** Root mean square fluctuation (RMSF) profiles highlighting localized residue flexibility. **(d)** Binding free energy curves estimated using the MM/PBSA-like approach implemented in YASARA.

Based on the network pharmacology results, AKT1 was selected as a representative candidate receptor protein for molecular docking. The docking results showed that DHM could be accommodated within the predicted binding pocket of AKT1, indicating a potential interaction between the two molecules. Visualization of the ligand-receptor interaction pattern further suggested relatively stable non-covalent interactions between DHM and AKT1 (Figure 4a).

Additional docking results showed that DHM also exhibited predicted binding potential with other hub targets, including EGF, EGFR, ESR1, IL6, MMP2, MMP9, and SRC, suggesting that DHM may have a multitarget-associated pharmacological profile. However, based on the network topology results and the subsequent experimental design, AKT1 was prioritized as the candidate target for further examination (Supplementary Figure S1).

To further assess the stability of the DHM-AKT1 complex under dynamic conditions, a 50-ns molecular dynamics simulation was performed on the optimal docking conformation. The RMSD curve of the DHM-AKT1 complex showed transient fluctuations during the initial phase of the simulation and then gradually reached a plateau, indicating that the overall conformation of the complex remained relatively stable (Figure 4b). RMSF analysis showed limited flexibility in most amino acid residues, with only a few local regions exhibiting relatively higher fluctuations (Figure 4c). In addition, the binding free energy profile suggested that DHM maintained a favorable predicted binding profile with AKT1 throughout the simulation (Figure 4d). Collectively, the molecular docking and molecular dynamics results supported the predicted stability of the DHM-AKT1 interaction model.

### 3.3 HS tissues and HSFs exhibited a fibrotic phenotype, and HSFs showed basal activation of PI3K/AKT/mTOR-related signaling

**FIGURE 5:**
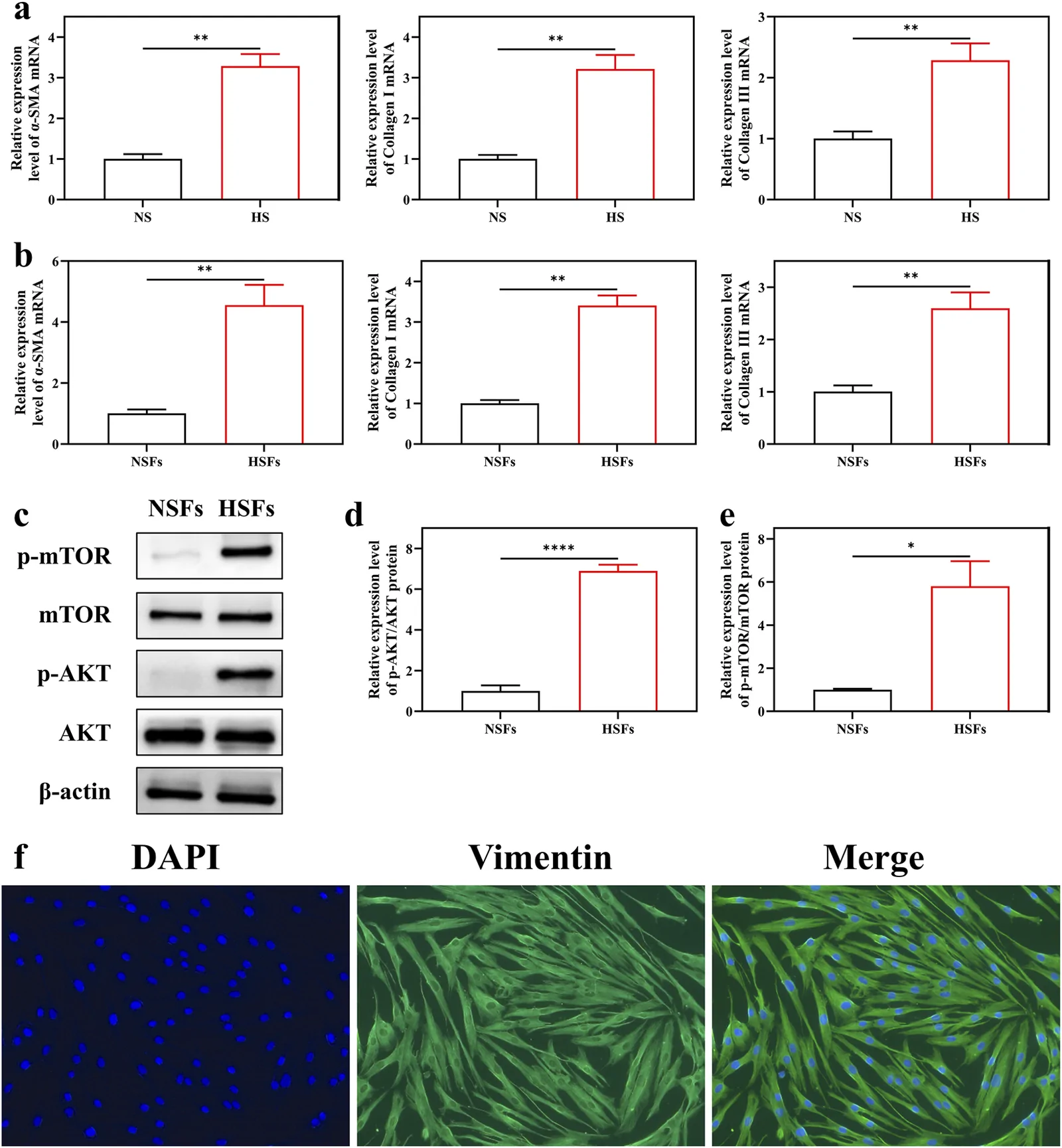
Characterization of HS tissues and fibroblasts and basal activation of PI3K/AKT/mTOR signaling in HSFs. **(a)** RT-qPCR analysis of Collagen I, Collagen III, and α-SMA mRNA expression in hypertrophic scar tissues and paired normal skin tissues. **(b)** RT-qPCR analysis of Collagen I, Collagen III, and α-SMA mRNA expression in hypertrophic scar fibroblasts and paired normal skin fibroblasts. **(c)** Representative Western blot bands of p-AKT, AKT, p-mTOR, and mTOR in HSFs and paired NSFs. **(d)** Quantitative analysis of the p-AKT/AKT ratio in HSFs and NSFs. **(e)** Quantitative analysis of the p-mTOR/mTOR ratio in HSFs and NSFs. **(f)** Representative immunofluorescence staining of primary fibroblasts for vimentin. More than 90% of cells were vimentin-positive, confirming the fibroblast phenotype. Data are presented as mean ± SD from three independent biological replicates. Statistical significance between paired groups was determined using paired-samples t tests. n = 3 per group.

To assess the characteristics of the clinical tissues and primary cell models used in this study, the expression of fibrosis-related molecules in hypertrophic scar tissues (HS tissues) and paired normal skin tissues (NS tissues) was first examined. RT-qPCR results showed that the expression levels of Collagen I, Collagen III, and α-SMA were significantly elevated in HS tissues relative to the paired NS tissues from the same patients, indicating marked fibrotic alterations and enhanced myofibroblast activation in hypertrophic scar tissue (Figure 5a).

At the cellular level, hypertrophic scar-derived fibroblasts (HSFs) were further compared with paired normal skin fibroblasts (NSFs) derived from the same donors. Compared with the paired NSFs, HSFs showed significantly higher expression levels of Collagen I, Collagen III, and α-SMA, indicating that the isolated and cultured HSFs retained the characteristic profibrotic phenotype of hypertrophic scar and were consistent with the *in vivo* pathological state (Figure 5b).

To further determine whether PI3K/AKT/mTOR-related signaling was basally activated in HSFs, the phosphorylation levels of AKT and mTOR were compared between untreated HSFs and paired NSFs. Western blot analysis showed that HSFs exhibited higher p-AKT/AKT and p-mTOR/mTOR ratios than paired NSFs, suggesting enhanced basal activation of PI3K/AKT/mTOR-related signaling in HSFs (Figures 5c–e). These findings provided cellular context for the subsequent examination of whether DHM treatment was accompanied by changes in PI3K/AKT/mTOR-related signaling.

Meanwhile, immunofluorescence staining for vimentin showed strong positive expression in the cultured cells, with more than 90% of cells being vimentin-positive, confirming their fibroblast identity (Figure 5f). Together, these results indicate that the clinical samples and primary fibroblast models used in this study exhibited typical fibrotic features of hypertrophic scar, and that HSFs showed increased basal PI3K/AKT/mTOR-related signaling compared with paired NSFs.

### 3.4 DHM reduced HSF metabolic viability and fibrosis-related marker expression with accompanying changes in PI3K/AKT/mTOR-related signaling

The effect of DHM on HSF viability was first evaluated using the CCK-8 assay. The results showed that DHM treatment for 24 h reduced the CCK-8 signal in a concentration-dependent manner, indicating decreased metabolic cell viability. Based on these findings, 0, 5, 10, and 20 μM DHM were used to evaluate dose-dependent changes in fibrosis-related markers and pathway-associated proteins. In addition, 15 μM DHM was selected as the working concentration for subsequent pathway-related experiments. To further assess whether this concentration induced overt acute cell death, live/dead staining was performed. The results did not show an obvious increase in PI-positive dead cells after 15 μM DHM treatment, suggesting that the decrease in CCK-8 signal may reflect reduced metabolic activity and/or growth inhibition rather than extensive acute cell death (Supplementary Figure S3).

**FIGURE 6:**
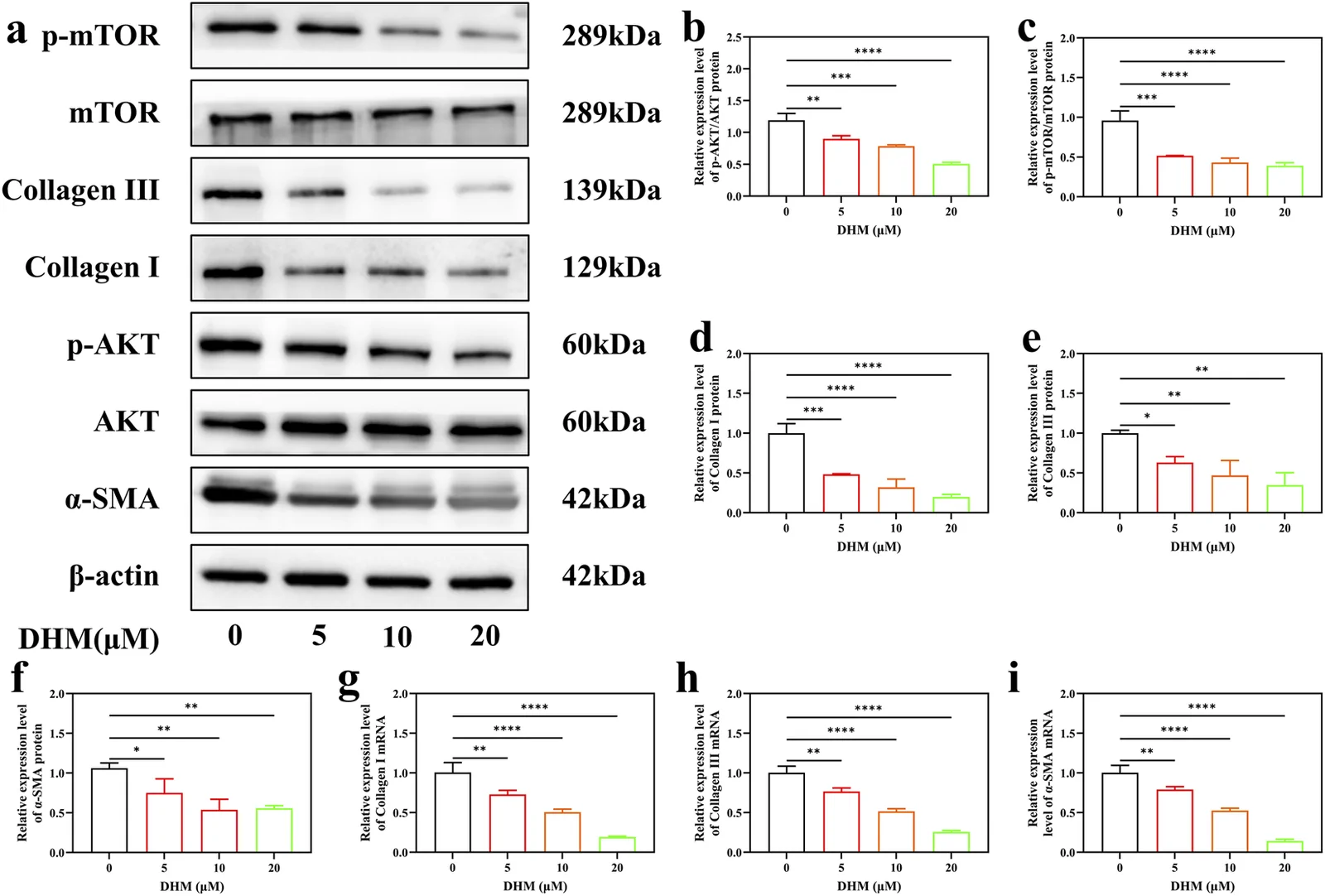
HSFs were treated with different concentrations of DHM. **(a)** Representative Western blot images. Quantitative analyses of **(b)** p-AKT/AKT, **(c)** p-mTOR/mTOR, **(d)** Collagen I/β-actin, **(e)** Collagen III/β-actin, and **(f)** α-SMA/β-actin are shown. RT-qPCR analyses further showed reduced mRNA expression of **(g)** Collagen I, **(h)** Collagen III, and **(i)** α-SMA. Data are presented as mean ± SD from three independent biological replicates. Statistical significance was determined using one-way ANOVA followed by Tukey’s multiple-comparisons test. n = 3 per group.

After treatment with different concentrations of DHM, Western blot analysis showed that the phosphorylation levels of AKT and mTOR in HSFs gradually decreased with increasing DHM concentration. At the same time, the protein expression levels of Collagen I, Collagen III, and α-SMA were also reduced, suggesting attenuation of fibrosis-associated features in HSFs (Figures 6a–f).

RT-qPCR further showed that the mRNA expression levels of Collagen I, Collagen III, and α-SMA were decreased after DHM treatment, showing an overall trend consistent with the protein-level results (Figures 6g–i).

In addition, supplementary results showed that the mRNA expression levels of AKT1 and mTOR in HSFs also decreased after DHM treatment, suggesting that DHM was accompanied by changes in PI3K/AKT/mTOR-related signaling at both the transcriptional and protein phosphorylation levels (Supplementary Figure S4). Taken together, these findings indicate that DHM reduced fibrosis-associated markers in HSFs and was accompanied by changes in PI3K/AKT/mTOR-related signaling. However, because DHM also reduced the CCK-8 viability signal, the observed decreases in fibrosis-related markers should be interpreted with caution, as they may reflect both attenuation of fibrosis-associated features and growth-inhibitory effects.

### 3.5 LY294002-alone and combined treatment further supported the involvement of PI3K/AKT/mTOR-related signaling in the effects of DHM

**FIGURE 7:**
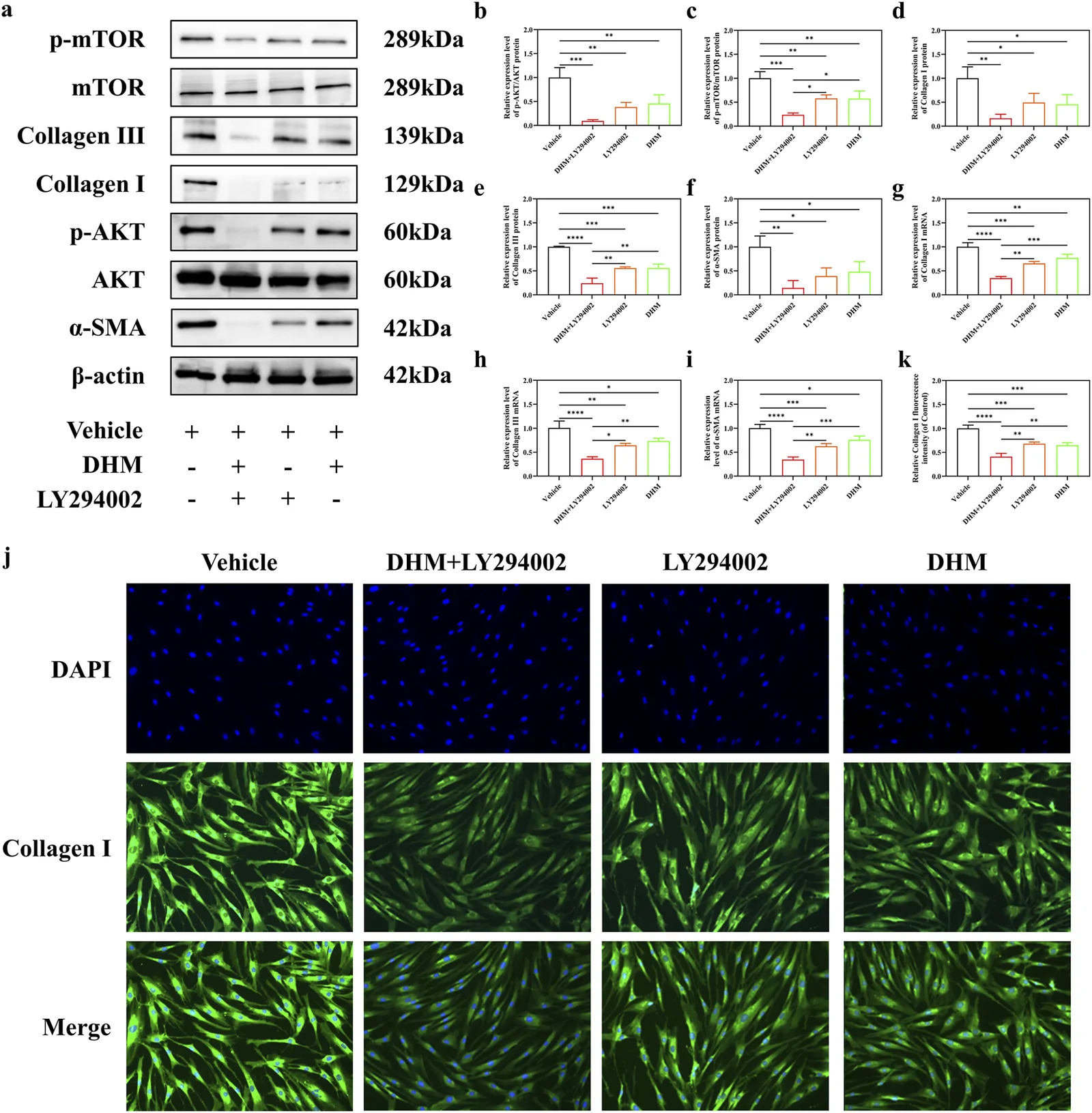
DHM attenuates fibrotic activation in HSFs with accompanying changes in PI3K/AKT/mTOR-related signaling. HSFs were treated with vehicle control, DHM alone, LY294002 alone, or DHM combined with LY294002 for 24 h. **(a)** Representative Western blot images of PI3K/AKT/mTOR-related signaling proteins and fibrosis-associated markers in HSFs. **(b–g)** Densitometric analysis of the relative protein expression levels of p-AKT/AKT, p-mTOR/mTOR, mTOR, Collagen I, Collagen III, and α-SMA. **(h,i)** RT-qPCR analysis of the relative mRNA expression levels of fibrosis-related genes in HSFs. **(j)** Representative immunofluorescence images of fibrosis-associated marker expression in HSFs. **(k)** Quantification of fluorescence intensity. Data are presented as mean ± SD from three independent biological replicates. Statistical significance was determined using one-way ANOVA followed by Tukey’s multiple-comparisons test. n = 3 per group.

To further examine the relationship between PI3K/AKT/mTOR-related signaling and the observed effects of DHM, HSFs were treated with vehicle control, DHM alone, LY294002 alone, or DHM combined with LY294002. Western blot results showed that, compared with the vehicle control group, DHM treatment reduced AKT and mTOR phosphorylation, accompanied by downregulation of Collagen I, Collagen III, and α-SMA protein expression in HSFs (Figures 7a–g). LY294002 alone also reduced AKT and mTOR phosphorylation and decreased fibrosis-related protein expression, supporting the involvement of PI3K/AKT/mTOR-related signaling in maintaining the fibrotic phenotype of HSFs.

Further comparison showed that the DHM + LY294002 group exhibited a greater reduction in AKT and mTOR phosphorylation and fibrosis-related protein expression than the DHM-alone group. These findings suggest that pharmacological inhibition of PI3K/AKT/mTOR-related signaling produced changes in the same direction as DHM treatment and further strengthened the DHM-associated reduction of fibrosis-related markers.

RT-qPCR results were generally consistent with the protein findings. Compared with the vehicle control group, the mRNA expression levels of Collagen I, Collagen III, and α-SMA were downregulated in the DHM group and the LY294002-alone group, while the reductions were more pronounced in the DHM + LY294002 group (Figures 7h,i). Supplementary data further showed that AKT and mTOR mRNA expression also decreased after LY294002 or DHM treatment and showed a greater decrease after combined treatment (Supplementary Figure S5).

Immunofluorescence results further supported these findings at the morphological level. Compared with the vehicle control group, Collagen I fluorescence intensity in HSFs was weakened after DHM treatment or LY294002 treatment, and this signal was further reduced after combined treatment with DHM and LY294002, suggesting reduced extracellular matrix-associated protein expression (Figures 7j,k). Taken together, the protein, mRNA, and immunofluorescence data provide pharmacological evidence supporting the involvement of PI3K/AKT/mTOR-related signaling in the DHM-associated attenuation of fibrosis-related features in HSFs. However, these findings should still be interpreted as supportive rather than definitive causal evidence because direct genetic manipulation, pathway activation, or rescue experiments were not performed.

### 3.6 DHM alleviated hypertrophic scar formation and reduced scar elevation and collagen deposition *in vivo*

**FIGURE 8:**
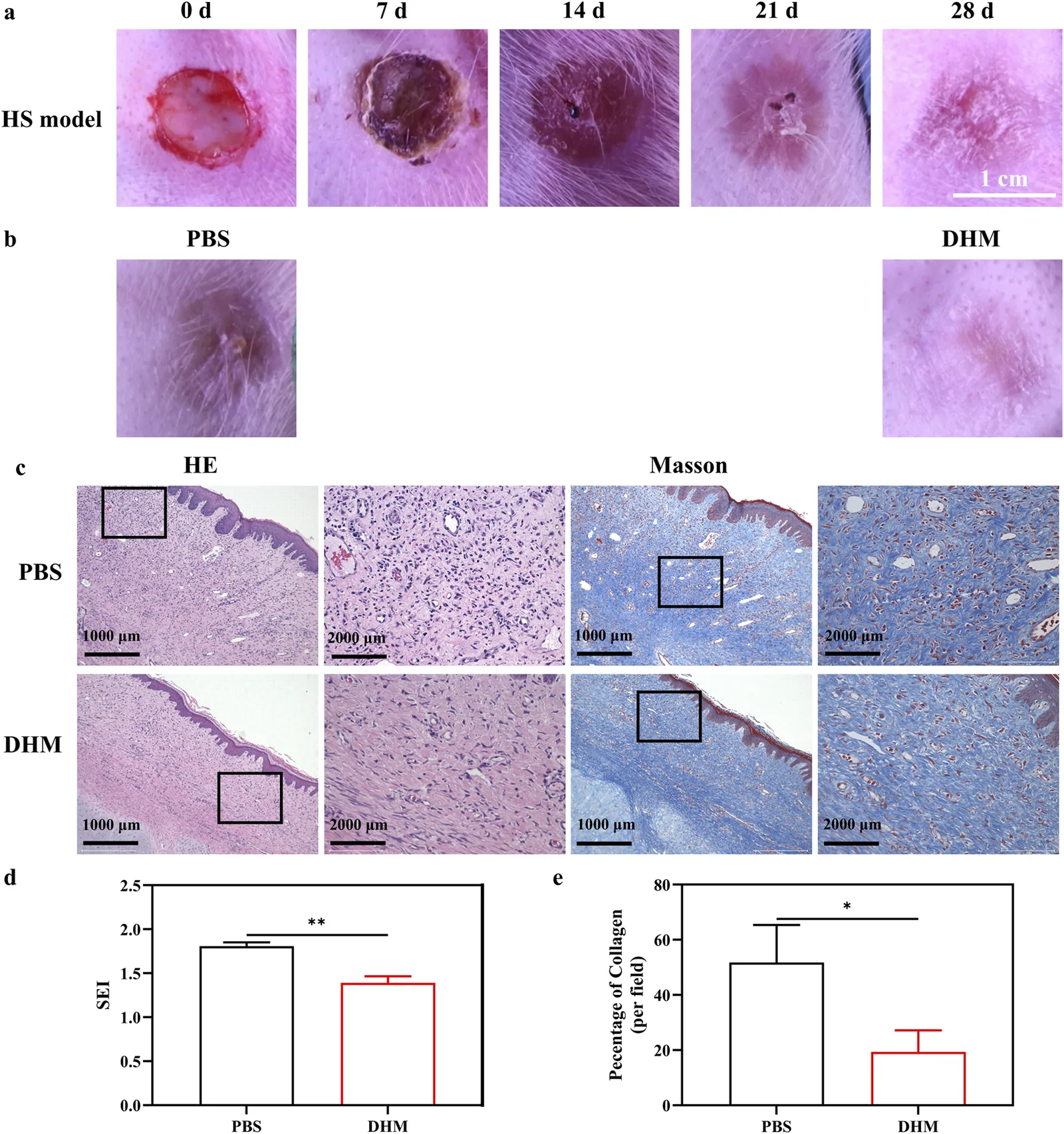
Gross morphological changes during rabbit ear hypertrophic scar formation and histological alterations after DHM treatment. **(a)** Representative gross images of hypertrophic scar formation on postoperative days 0, 7, 14, 21, and 28. **(b)** Representative gross appearance of scars in the PBS and DHM groups after 28 days of treatment. **(c)** Representative histological images of scars after 28 days of treatment. Upper row: PBS group; lower row: DHM group. From left to right in each row: low- and high-magnification H&E staining, and low- and high-magnification Masson’s trichrome staining. Scale bars as indicated. **(d)** Quantitative analysis of the scar elevation index (SEI) in the PBS and DHM groups. **(e)** Quantitative analysis of collagen deposition based on the collagen-positive area fraction in Masson’s trichrome-stained sections. Data are presented as mean ± SD. SEI and collagen-positive area fraction were quantified from three histological sections for each treatment condition (n = 3 sections). Each section was analyzed as one statistical unit. Because PBS- and DHM-treated samples were obtained under a paired experimental design, statistical significance was determined using paired-samples t tests.

To assess the anti-scar effect of DHM *in vivo*, a rabbit ear hypertrophic scar model was established, and wound healing and scar formation were dynamically observed. A total of 48 wounds were created in six rabbits. On postoperative day 7, scabs began to form on the wound surface and appeared grayish-brown. By day 14, most wounds had completed re-epithelialization. By day 21, re-epithelialization was nearly complete and local induration could be palpated. By day 28, scar hyperplasia reached its peak, forming typical rabbit ear hypertrophic scars characterized by obvious local elevation, firm texture, and reddish appearance, while the hyperplastic area did not extend beyond the original wound margin. The scar thickness was approximately 2.5 times that of normal ear tissue (Figure 8a). Among the 48 wounds, 30 developed obvious raised scar-like tissue above the skin surface, corresponding to a scar formation rate of 62.5%, indicating successful establishment of the rabbit ear hypertrophic scar model.

In the subsequent intervention experiment, the gross appearance of scars was improved in the DHM-treated group compared with the paired PBS-treated scars from the same animals. After 28 days of treatment, scars in the PBS group still showed obvious local elevation, firm texture, and reddish color, whereas scar hyperplasia in the DHM group was alleviated, with a flatter appearance, softer texture, and paler color (Figure 8b). Quantitative analysis further showed that the scar elevation index (SEI) was significantly reduced in the DHM group compared with the paired PBS group, indicating that DHM decreased scar elevation in the rabbit ear hypertrophic scar model (Figure 8d).

Histological results were consistent with the gross observations. H&E staining showed that scar tissues in the PBS group exhibited marked epidermal thickening, inflammatory cell infiltration, fibroblast proliferation, and neovascularization in the dermis. In contrast, these pathological changes were alleviated after DHM treatment. Masson’s trichrome staining showed that collagen fibers were excessively deposited in the PBS group and displayed a disorganized, nodular, or whorled arrangement. By contrast, collagen deposition appeared reduced in the DHM group, and the collagen fibers were more orderly and relatively loose (Figure 8c). Consistently, quantitative analysis of Masson’s trichrome-stained sections showed that the collagen-positive area fraction was significantly lower in the DHM group than in the paired PBS group (Figure 8e). Together, these gross morphological, histological, and quantitative findings indicate that DHM alleviated scar-associated fibrotic features in the rabbit ear hypertrophic scar model.

## 4 Discussion

Hypertrophic scar is a common pathological fibrotic outcome during wound repair and is characterized by abnormal fibroblast activation, enhanced myofibroblast differentiation, and excessive extracellular matrix deposition (Mony et al., 2023; Kohlhauser et al., 2024). Collagen I, Collagen III, and α-SMA are widely used indicators of fibrotic activation in hypertrophic scar, while several signaling pathways, including TGF-β/Smad, MAPK, and PI3K/AKT/mTOR-related signaling, have been implicated in fibroblast proliferation, differentiation, survival, and collagen synthesis (Mony et al., 2023; Zhao et al., 2024; Dong et al., 2024; Li et al., 2024; Fan et al., 2020). Among these pathways, TGF-β/Smad signaling is generally considered a central profibrotic pathway in scar formation. However, hypertrophic scar fibrosis is not regulated by a single linear pathway. PI3K/AKT/mTOR-related signaling may interact with TGF-β/Smad-associated responses and contribute to the maintenance of fibroblast activation and extracellular matrix accumulation. Therefore, investigating PI3K/AKT/mTOR-related signaling provides one relevant perspective for understanding the anti-fibrotic effects of candidate compounds in hypertrophic scar.

In the present study, the potential anti-scar effect of DHM on hypertrophic scar was investigated using an integrated computational and experimental framework, including network pharmacology, molecular docking, molecular dynamics simulation, tissue- and cell-level validation, and an animal model. The results showed that the overlapping targets of DHM and hypertrophic scar formed a significantly enriched interaction network in the PPI analysis. AKT1 was identified as one of the common hub target candidates and was prioritized for further examination because of its central position in the network, its relevance to PI3K/AKT-related enrichment results, and its biological association with fibroblast activation and fibrosis-related signaling. Molecular docking and 50-ns molecular dynamics simulation supported a predicted favorable interaction pattern between DHM and AKT1. Experimentally, HS tissues and HSFs showed increased expression of Collagen I, Collagen III, and α-SMA, and untreated HSFs exhibited higher basal p-AKT/AKT and p-mTOR/mTOR ratios than paired NSFs. DHM treatment reduced fibrosis-associated markers in HSFs and was accompanied by decreased AKT and mTOR phosphorylation. In the rabbit ear hypertrophic scar model, DHM improved gross scar appearance, reduced scar elevation, decreased collagen-positive area fraction, and alleviated histopathological alterations. Collectively, these findings indicate that DHM attenuated fibrosis-associated features of hypertrophic scar *in vitro* and *in vivo*, and that these effects were accompanied by changes in PI3K/AKT/mTOR-related signaling.

Network pharmacology provided a useful hypothesis-generating framework for this study (Lee et al., 2025; Panossian, 2025). Compared with the traditional single-target strategies, network pharmacology better reflects the multi-target and multi-pathway characteristics of natural compounds (Lee et al., 2025; Panossian, 2025). In the present study, the overlapping targets related to DHM exhibited a high interaction density in the PPI network, and GO and KEGG enrichment analyses suggested associations with cell proliferation, migration, extracellular matrix remodeling, and PI3K/AKT-related signaling pathways. These findings suggest that the potential effects of DHM on hypertrophic scar are unlikely to depend on a single isolated molecule, but may involve a regulatory network of multiple functionally related targets. In addition to AKT1, other hub target candidates, including EGFR, SRC, MMP9, MMP2, IL6, EGF, and ESR1, were also identified. Therefore, AKT1 should be regarded as a prioritized candidate target rather than the only potential target of DHM. This interpretation is also consistent with the multi-target pharmacological characteristics of natural flavonoids.

The molecular docking and molecular dynamics results provided structural support for the predicted interaction between DHM and AKT1. Docking analysis suggested that DHM could be accommodated within the predicted receptor pocket of AKT1, while molecular dynamics simulation showed that the RMSD of the DHM-AKT1 complex gradually stabilized during the simulation, the overall RMSF remained relatively low, and the binding free energy stayed within a favorable range. These computational findings do not constitute direct evidence of target engagement in cells or tissues, but they support the predicted stability of the DHM-AKT1 interaction model and provide a basis for subsequent experimental evaluation (Lee et al., 2025).

At the tissue and cellular levels, the reliability of the experimental models was first assessed in this study. Compared with paired NS tissues and NSFs, HS tissues and HSFs showed increased expression of Collagen I, Collagen III, and α-SMA, indicating that the clinical samples and primary cell models exhibited characteristic fibrotic features of hypertrophic scar (Mony et al., 2023). Vimentin immunofluorescence further confirmed the fibroblast phenotype of the cultured cells. Importantly, untreated HSFs also showed higher p-AKT/AKT and p-mTOR/mTOR ratios than paired NSFs, suggesting basal activation of PI3K/AKT/mTOR-related signaling in HSFs. This finding provides pathological context for the subsequent observation that DHM treatment was accompanied by reduced AKT and mTOR phosphorylation.

The *in vitro* experiments provided an important line of evidence for the anti-fibrotic activity of DHM. CCK-8 results showed that DHM reduced the viability signal of HSFs in a concentration-dependent manner. Further Western blotting and RT-qPCR analyses demonstrated that DHM treatment reduced the protein and mRNA expression levels of Collagen I, Collagen III, and α-SMA in HSFs, suggesting attenuation of the fibrotic activation state of fibroblasts. Because α-SMA is a commonly used marker of myofibroblast differentiation, its reduction is consistent with a less activated fibrotic phenotype (Mony et al., 2023). However, the reduction in CCK-8 signal should be interpreted carefully. CCK-8 primarily reflects metabolic activity and does not by itself distinguish between reduced proliferation, altered metabolism, and cell death. In this study, live/dead staining did not show an obvious increase in PI-positive dead cells after 15 μM DHM treatment, suggesting that the decrease in CCK-8 signal may reflect reduced metabolic activity and/or growth inhibition rather than extensive acute cell death. Nevertheless, the possibility that growth-inhibitory effects contributed to the reduction in fibrosis-related markers cannot be fully excluded.

Changes in PI3K/AKT/mTOR-related signaling were observed at multiple levels. With increasing concentrations of DHM, AKT and mTOR phosphorylation gradually decreased, and supplementary experiments further showed reduced AKT and mTOR mRNA expression after DHM treatment. These findings suggest that DHM attenuated fibrosis-related features in HSFs and that these effects were accompanied by changes in PI3K/AKT/mTOR-related signaling (Zhao et al., 2024; Dong et al., 2024). However, because DHM also reduced the CCK-8 viability signal and because direct genetic manipulation, pathway activation, or rescue experiments were not performed, the current data are more appropriate for supporting pathway involvement rather than establishing a definitive fibrosis-specific or causal mechanism (Panossian, 2025).

To further examine the relationship between PI3K/AKT/mTOR-related signaling and the observed effects of DHM, LY294002 was introduced as a pharmacological inhibitor. The LY294002-alone group reduced AKT and mTOR phosphorylation and fibrosis-related marker expression, supporting the involvement of PI3K/AKT/mTOR-related signaling in maintaining the fibrotic phenotype of HSFs. Moreover, the DHM + LY294002 group showed more pronounced reductions in AKT and mTOR phosphorylation, Collagen I, Collagen III, and α-SMA expression than the DHM-alone group. Immunofluorescence analysis also demonstrated a further decrease in Collagen I fluorescence intensity after combined treatment. These findings provide pharmacological evidence supporting the involvement of PI3K/AKT/mTOR-related signaling in the DHM-associated attenuation of fibrosis-related features in HSFs. Nevertheless, these data should still not be interpreted as definitive mechanistic proof (Zhao et al., 2024; Dong et al., 2024; Lee et al., 2025). A pathway activator, AKT1 overexpression, AKT1 knockdown, or rescue assay would be required to determine whether the anti-fibrotic effects of DHM are dependent on this signaling axis.

The relationship between PI3K/AKT/mTOR-related signaling and TGF-β/Smad signaling also deserves consideration. TGF-β/Smad signaling is one of the most extensively studied profibrotic pathways in hypertrophic scar and is closely associated with myofibroblast differentiation and ECM deposition (Mony et al., 2023; Zhao et al., 2024; Dong et al., 2024). PI3K/AKT/mTOR-related signaling may function as a parallel or interacting pathway that influences fibroblast survival, proliferation, metabolism, and collagen synthesis. Therefore, the DHM-associated reduction in AKT and mTOR phosphorylation observed in this study may represent one component of a broader anti-fibrotic signaling response rather than a single exclusive mechanism. Future studies should examine whether DHM also modulates TGF-β/Smad signaling and whether crosstalk between TGF-β/Smad and PI3K/AKT/mTOR contributes to the regulation of HSF activation.

In addition to the *in vitro* experiments, the present study examined the *in vivo* effect of DHM using a rabbit ear hypertrophic scar model. The gross appearance of scars was improved in the DHM-treated group compared with the paired PBS group, with reduced local hyperplasia. H&E staining indicated that epidermal thickening, inflammatory cell infiltration, fibroblast proliferation, and neovascularization were alleviated after DHM treatment. Masson’s trichrome staining further showed reduced collagen deposition and a more orderly collagen arrangement. Importantly, quantitative analyses showed that DHM reduced the scar elevation index and decreased the collagen-positive area fraction in Masson’s trichrome-stained sections. These quantitative results strengthen the *in vivo* evidence and indicate that the anti-fibrotic effect of DHM is not limited to the cell culture system but also has biological relevance in the rabbit ear hypertrophic scar model (Dong et al., 2024; Zhang et al., 2024).

Nevertheless, this study still has several limitations. First, network pharmacology is essentially a database-based predictive approach, and its results are influenced by database completeness, target annotation, and screening thresholds (Lee et al., 2025; Panossian, 2025). Second, although AKT1 was prioritized as a hub target candidate and DHM treatment was accompanied by changes in PI3K/AKT/mTOR-related signaling, the current study did not include direct target-engagement assays, AKT1 knockdown or overexpression, pathway activation, or rescue experiments. Therefore, the present data support pathway involvement but do not establish a definitive causal mechanism. Third, although live/dead staining did not indicate extensive acute cell death after 15 μM DHM treatment, DHM reduced the CCK-8 viability signal; therefore, growth inhibition or altered metabolic activity may have partly contributed to the observed reductions in fibrosis-related markers. Fourth, the clinical sample size was limited, and patient age, scar site, and individual biological variability may have influenced fibroblast behavior. Fifth, although SEI and collagen-positive area fraction were added to strengthen the animal data, hydroxyproline measurement and tissue-level Western blotting or immunohistochemistry were not performed. These additional analyses would further strengthen the *in vivo* mechanistic evidence. Sixth, although DHM showed promising anti-fibrotic effects in HSFs and in the rabbit ear hypertrophic scar model, further translational evaluation is required before considering its development as a topical anti-scar candidate. The present study did not assess skin penetration, local drug retention, optimal topical formulation, dose-response relationships *in vivo*, long-term biosafety, or potential local irritation and sensitization after repeated administration. These issues should be addressed in future studies to better evaluate the translational potential of DHM for hypertrophic scar management.

In conclusion, this study showed that DHM attenuated fibrosis-associated features of hypertrophic scar *in vitro* and *in vivo*. Computational prediction, pharmacological inhibition, cellular experiments, and animal results together indicate that these observed effects were accompanied by changes in PI3K/AKT/mTOR-related signaling. Although the current findings do not establish a definitive causal regulatory relationship, they support the involvement of PI3K/AKT/mTOR-related signaling in the DHM-associated attenuation of fibrosis-related features and suggest that DHM may represent a potential candidate for further anti-scar investigation.

## Data Availability

The raw data supporting the conclusions of this article will be made available by the authors, without undue reservation.
