# Supplementary material for: Dihydromyricetin attenuates fibrosis-associated features of hypertrophic scar with accompanying changes in PI3K/AKT/mTOR-related signaling
Source: Front Pharmacol. 2026 Aug 12;17:1862651. doi: 10.3389/fphar.2026.1862651 (PMC13506778; doi:10.3389/fphar.2026.1862651)
Supplement: Supplementary file 1 [file Table4.docx]

**Supplementary Materials**

**Figure S1.** Molecular docking of DHM with hub target candidates in hypertrophic scar.
Visualization of the predicted molecular docking patterns between DHM and the selected hub target candidates, including EGF, EGFR, ESR1, IL6, MMP2, MMP9, and SRC.

**Figure S2.**  KEGG pathway map showing PI3K/AKT signaling-related targets among the overlapping targets.

**Figure S3.** Effects of dihydromyricetin on HSF viability and cell survival. **(a)** HSF viability was assessed using the CCK-8 assay after treatment with different concentrations of dihydromyricetin for 24 h. Data are presented as mean ± SD from four independent biological replicates (n = 4). **(b)** Representative live/dead staining images of HSFs after treatment with 15 μM dihydromyricetin for 24 h. The live/dead staining experiment was repeated independently three times (n = 3) with similar results.

**Figure S4.** Effects of DHM on AKT and mTOR mRNA expression in hypertrophic scar fibroblasts. **(a)** Relative AKT mRNA expression. **(b)** Relative mTOR mRNA expression. Data are presented as mean ± SD from three independent biological replicates. Statistical significance was determined using one-way ANOVA followed by Tukey’s multiple-comparisons test. n = 3 per group.

**Figure S5.** Effects of DHM and LY294002 on AKT and mTOR mRNA expression in hypertrophic scar fibroblasts. **(a)** Relative AKT mRNA expression. **(b)** Relative mTOR mRNA expression. Data are presented as mean ± SD from three independent biological replicates. Statistical significance was determined using one-way ANOVA followed by Tukey’s multiple-comparisons test. n = 3 per group.

**Supplementary** **Table 1** Clinical characteristics of patients and paired tissue samples.

| Patient ID | Duration（months） | Sex | Age（years） | HS side | NS side |
| --- | --- | --- | --- | --- | --- |
| D71602 | 6 | Male | 25 | Shoulder | Arm |
| D57635 | 10 | Male | 18 | Chest | Abdomen |
| E25378 | 8 | Female | 12 | Chest | Arm |
| D93561 | 7 | Female | 27 | Neck | Abdomen |
| D54234 | 8 | Female | 43 | Back | Back |
| D54231 | 7 | Male | 36 | Buttock | Abdomen |

**Supplementary** **Table 2** List of primer sequences

| Gene name | Direction | Primer Sequences (5’ to 3’) |
| --- | --- | --- |
| GAPDH | Forward (F) | GGA GCG AGA TCC CTC CAA AA |
|  | Reverse (R) | GGC TGT TGT CAT ACT TCT CAT GG |
| AKT | Forward (F) | CTG GTC CTG TCT TCC TCA TGT T |
|  | Reverse (R) | AGG CAG CCC CTT TGA CTT CT |
| mTOR | Forward (F) | GCT TAG AGG ACA GCG GGG AA |
|  | Reverse (R) | AAG CAT CTT GCC CTG AGG TT |
| Collagen I | Forward (F) | GAG GGC CAA GAC GAA GAC ATC |
|  | Reverse (R) | CAG ATC ACG TCA TCG CAC AAC |
| Collagen III | Forward (F) | GGA GCT GGC TAC TTC TCG C |
|  | Reverse (R) | GGG AAC ATC CTC CTT CAA CAG |
| **α-SMA** | Forward (F) | AAA AGA CAG CTA CGT GGG TGA |
|  | Reverse (R) | GCC ATG TTC TAT CGG GTA CTT C |
